# Precise pretreatment of lignocellulose: relating substrate modification with subsequent hydrolysis and fermentation to products and by-products

**DOI:** 10.1186/s13068-017-0775-3

**Published:** 2017-04-11

**Authors:** Fan Lü, Lina Chai, Liming Shao, Pinjing He

**Affiliations:** 1 State Key Laboratory of Pollution Control and Resource Reuse, Tongji University, Shanghai, 200092 China; 2 Institute of Waste Treatment and Reclamation, Tongji University, Shanghai, 200092 China; 3 Centre for the Technology Research and Training on Household Waste in Small Towns & Rural Area, Ministry of Housing and Urban–Rural Development (MOHURD) of China, Shanghai, 200092 China

**Keywords:** Lignocellulose, Precise pretreatment, Selective pretreatment, Environmental assessment, Crystalline form, Holistic view

## Abstract

**Background:**

Pretreatment is a crucial step for valorization of lignocellulosic biomass into valuable products such as H_2_, ethanol, acids, and methane. As pretreatment can change several decisive factors concurrently, it is difficult to predict its effectiveness. Furthermore, the effectiveness of pretreatments is usually assessed by enzymatic digestibility or merely according to the yield of the target fermentation products. The present study proposed the concept of “precise pretreatment,” distinguished the major decisive factors of lignocellulosic materials by precise pretreatment, and evaluated the complete profile of all fermentation products and by-products. In brief, hemicellulose and lignin were selectively removed from dewaxed rice straw, and the cellulose was further modified to alter the crystalline allomorphs. The subsequent fermentation performance of the selectively pretreated lignocellulose was assessed using the cellulolytic, ethanologenic, and hydrogenetic *Clostridium thermocellum* through a holistic characterization of the liquid, solid, and gaseous products and residues.

**Results:**

The transformation of crystalline cellulose forms from I to II and from *I*_*α*_ to *I*_*β*_ improved the production of H_2_ and ethanol by 65 and 29%, respectively. At the same time, the hydrolysis efficiency was merely improved by 10%, revealing that the crystalline forms not only influenced the accessibility of cellulose but also affected the metabolic preferences and flux of the system. The fermentation efficiency was independent of the specific surface area and degree of polymerization. Furthermore, the pretreatments resulted in 43–45% of the carbon in the liquid hydrolysates unexplainable by forming ethanol and acetate products. A tandem pretreatment with peracetic acid and alkali improved ethanol production by 45.5%, but also increased the production of non-ethanolic low-value by-products by 136%, resulting in a huge burden on wastewater treatment requirements.

**Conclusion:**

Cellulose allomorphs significantly affected fermentation metabolic pathway, except for hydrolysis efficiency. Furthermore, with the increasing effectiveness of the pretreatment for ethanol production, more non-ethanolic low-value by-products or contaminants were produced, intensifying environmental burden. Therefore, the effectiveness of the pretreatment should not only be determined on the basis of energy auditing and inhibitors generated, but should also be assessed in terms of the environmental benefits of the whole integrated system from a holistic view.

**Electronic supplementary material:**

The online version of this article (doi:10.1186/s13068-017-0775-3) contains supplementary material, which is available to authorized users.

## Background

Lignocellulosic biomass is an important renewable resource for the bioproduction of ethanol, H_2_, or organic acids [1, 2]. Numerous studies have established the importance of delignification, depolymerization, and decrystallization by various pretreatments [3–9]. The contents of lignin and hemicellulose, the degrees of crystallinity and polymerization, and the accessibility of the cellulose are regarded as the decisive factors affecting the effectiveness of pretreatments [10–14]. Since a pretreatment can change the above-mentioned parameters concurrently, the exact mechanism(s) underlying the effectiveness of diverse pretreatment strategies under different reaction conditions on diverse lignocellulosic materials are not conclusively known [3, 10, 12, 15].

Meanwhile, the effectiveness of pretreatments is invariably assessed by enzymatic hydrolysis or digestibility [11, 16–21], or according to the yield of the target fermentation products (mainly ethanol) [22]. Higher digestibility is preferred, while the fate of hydrolysates or non-target fermentation products from the pretreated biomass is seldom considered. Factors such as whether or not and how pretreatments affect a subsequent microbial fermentation are seldom discussed or included with the several potential mechanisms. If hydrolysis is increased and fermentation is decreased by pretreatment, the reduced fermentation efficiency is often attributed to the formation of inhibitory by-products during pretreatment [6].

Accordingly, the possibility of a precise pretreatment on lignocellulose is then speculated. Precise pretreatment is defined as the pretreatment measures that are carefully selected, optimized, combined, and customized, precisely according to the modification mechanism of each measure and the potential effect on the subsequent product-oriented utilization; the potential effect of each measure is fully expectable and controllable.

In targeting the question of how pretreatment of lignocelluloses affects the subsequent fermentation performance, in the present study the hemicellulose or lignin was selectively removed from the lignocellulose, and the crystalline allomorph of the cellulose was further modified. The selectively pretreated lignocelluloses were subjected to fermentation by anaerobic *Clostridium thermocellum*, realizing simultaneous solid-state saccharification and production of ethanol, H_2_, and acids. A holistic characterization of the pretreated substrates and the liquid, solid, and gaseous products and residues was conducted, aiming at comprehensively revealing the efficiency through precise pretreatment.

## Methods

### Raw materials and anaerobic microorganism

Fresh rice straw was sequentially washed with distilled water, air-dried, dewaxed with toluene/ethanol (2:1, v/v) at 150 °C for 6 h in an auto-Soxhlet apparatus (Soxtec™ 2050, FOSS, Denmark), washed with ethanol until the residual toluene was thoroughly removed, and then dried at 35 °C in an oven to constant weight. The dewaxed rice straw sample was denoted as “S.”

The thermophilic anaerobic cellulolytic *C. thermocellum* strain DSM 2360 was obtained from DSMZ (Deutsche Sammlung von Mikroorganismen und Zellkulturen, Germany) and cultivated as described previously [23, 24].

### Pretreatment measures

Three types of pretreatment were applied to the rice straw prepared as described above. (1) Dilute acid pretreatment was used to remove hemicellulose and increase the porosity (sample denoted as “DS”): the dewaxed straw was mixed with 1.5% (w/v) dilute sulfuric acid at a solid-to-liquid ratio of 2.5% (w/v) at 121 °C for 1 h. (2) Peracetic acid (PAA) pretreatment was used to remove lignin (sample denoted as “PS”): the dewaxed straw was mixed with PAA at a solid-to-liquid ratio of 1:10 (w/v) at 80 °C for 2 or 3 h (samples denoted as “PS2’’ and “PS3,” respectively). (3) Tandem PAA-NaOH pretreatment was used to remove lignin and modify the cellulose (sample denoted as “NS”): the above PAA-pretreated (2 h) straw was mercerized by 12.5% (w/v) NaOH at a solid-to-liquid ratio of 1:20 (w/v) at 30 °C for 1.5 h. The pretreated rice straw samples were washed thoroughly with hot distilled water until the eluate was pH neutral, and then dried at 35 °C to constant weight.

### Anaerobic fermentation of rice straw

The pretreated and untreated rice straw samples served as substrates for *C. thermocellum*. The incubation was conducted in triplicate at 55 ± 2 °C in a 250-mL serum bottle containing 1.5 g rice straw, 150 mL cultivation medium, and 7.5 mL of freshly harvested *C. thermocellum*, and sparged with N_2_ to obtain anaerobic status. Meanwhile, a blank without substrate that only contained *C. thermocellum* broth was used as the control. During the incubation, on the 461st h, the pH value of all the batches was regulated to 7.2 with the addition of NaOH and HCl solutions under anaerobic condition.

Liquid samples were collected periodically during each run, under anaerobic conditions, and the pH, volatile fatty acids, alcohols, and dissolved organic carbon (DOC) were measured. The production of gaseous H_2_ was calculated by determining the pressure and composition of the gas in the headspace.

### Analysis of liquid and gaseous metabolites from anaerobic fermentation

The pH was measured immediately after liquid sampling using a pH meter (pHS-2F, Shanghai Precision and Scientific Instrument Co. Ltd. China). After centrifugation at 16,000×*g* for 10 min, the contents of alcohols (including methanol, ethanol, *n*-propanol, *i*-propanol, *n*-butanol) and acids (including acetic, propionic, *n*-butyric, isobutyric, isovaleric, and *n*-valeric acids) in the supernatants of the liquid samples were determined by gas chromatography (6890N-FID, Agilent, USA) using a system equipped with a DB-WAXETR1 capillary column (30 m × 0.53 mm i.d. × 1 µm) according to the protocol described previously [24]. The DOC of the metabolites was detected using a total organic carbon analyzer (TOC V-CPN, Shimadzu, Japan). The gaseous composition (H_2_, CO_2_, CH_4_, N_2_, O_2_) and pressure were detected by gas chromatography (GC9800, Shanghai Precision and Scientific Instrument Co. Ltd. China) and using a pressure meter (TESTO 512, Barometer, Germany), respectively.

### Biochemical components and functional groups of the fibers

The cellulose, hemicellulose, and lignin contents in the fiber samples were determined by sequentially extracting the neutral detergent fiber, acid detergent fiber, lignin, and ash, according to the modified method proposed previously [25, 26]. The sequential extraction was conducted in a crude fiber extractor (FiberCap™ 2021, FOSS, Denmark).

The functional groups on the surface of the fibers were detected using an X-ray photoelectron spectrometer (XPS, PHI 5000C ESCA). The area illuminated by the irradiation was <1 mm in diameter. XPS wide scans at 0–1100 eV and C1s core-level spectra were recorded with the steps of 1.0 and 0.15 eV, respectively.

### Crystallinity and crystal forms of cellulose in the fibers

The crystallinity and crystal forms of the cellulose were characterized by solid-state cross-polarization/magic angle spinning (CP/MAS) ^13^C NMR. Cellulose was isolated according to the following protocol, as modified from a previous procedure [26]; Briefly, the fiber samples (9 g) were dispersed into 750 mL of deionized water. Then glacial acetic acid (6 mL) and sodium chlorite (6 g) were added to the mixture. The mixture was sealed in a reaction flask and maintained at 70 °C with stirring for 2 h. This treatment was repeated until the solid residue turned white and the lignin content was very low. After that, cellulose was isolated from 2 g of solid residue by soaking in 200 mL of 2.5 M HCl at 100 °C for 4 h and was then filtered and washed with deionized water.

Solid-state NMR measurements were carried out on a AVANCE III 400 WB spectrometer (Bruker, Germany) operating at a frequency of 100.69 MHz for ^13^C using a 4-mm Bruker double-resonance MAS probe head at a spinning speed of 10 kHz. Acquisition was performed with a CP pulse sequence using a 2.5-µs proton 90° pulse, a 1.5-ms contact pulse, and a 3.0-s delay between repetitions.

### Degree of polymerization of cellulose in the fibers

The number-average molecular weight ($\bar{M}_{\text{n}}$) and the weight-average molecular weight ($\bar{M}_{\text{w}}$) were determined by gel permeation chromatography after tricarbanilation of the cellulose according to a previously described procedure [27]. Gel permeation chromatography (Waters 515, Milford, MA, USA) equipped with three columns, Styragel HR3, HR4, and HR5, and a refractive index detector was run at a column temperature of 35 °C and a flow rate of 1 mg/mL. The weight-average degree of polymerization (DP_w_) and the number-average degree of polymerization (DP_n_) were obtained by dividing $\bar{M}_{\text{w}}$ and $\bar{M}_{\text{n}}$, respectively, by 519 g/mol, i.e., the molecular weight of the cellulose tricarbanilate monomer.

### Specific surface area and micro-morphological structure

The specific surface area of the fibers was determined by Brunauer, Emmett, and Teller (BET) nitrogen adsorption–desorption isotherms at −195 °C in a surface area analyzer (ASAP 2020 V3.04H, Micromeritics Co. Norcross, GA, USA). Prior to determination, the sample was degassed for 3 h at 80 °C under vacuum (*P*/*P*_0_ = 0.25) to remove moisture and any other contaminants.

The surface micro-morphological structures of the exterior and interior surfaces of the samples were examined by scanning electron microscopy (SEM, S-3400 N, Hitachi Ltd. Tokyo, Japan) at a voltage of 15 kV. Specimens were prepared for SEM inspection by placing the samples on carbon glue and then plating them with Pt (7 nm). Ultrathin sections of fibers were utilized to observe the changes in cell walls detected by transmission electron microscopy (TEM) and atomic force microscopy (AFM). TEM was conducted on a JEM-2100 microscope (JEOL, Tokyo, Japan). AFM was conducted on a Dimension 3100 microscope (Veeco Instruments Inc. Plainview, NY, USA). Images were captured using silicone cantilevers. The scanning rate ranged from 0.5 to 1.5 Hz.

### In situ visualization of cellulose accessibility to cellulase

The accessibility of the cellulose to cellulase was detected by Trx-Green Fluorescent Protein-CtCBM3 (TGC) labeling and observed by confocal laser scanning microscopy (Leica TCS SP5II, Leica, Mannheim, Germany) under 63 × 10 magnification. The details of TGC labeling were described previously [23], where bovine serum albumin (BSA) protein was used to block the lignin. A 488-nm argon laser was used for excitation. The emission bandwidth was set between 500 and 550 nm for collection of the TGC fluorescence. Images were obtained within a 30-µm *z*-depth containing eight confocal planes and obtained at a resolution of 1024 × 1024 pixels.

## Results

### Metabolite recovery via anaerobic fermentation

**Fig. 1:**
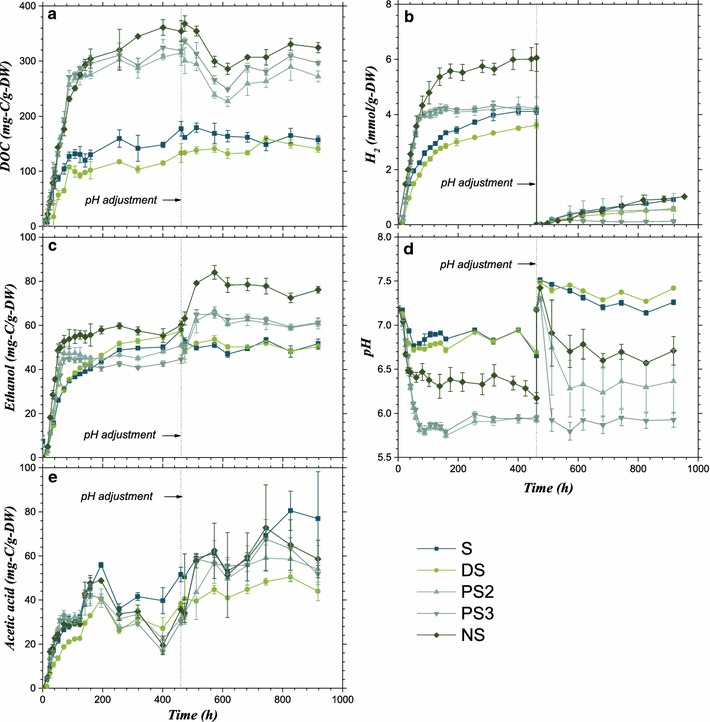
Yields of metabolites from anaerobic fermentation of untreated and pretreated straw. **a** Dissolved organic carbon, **b** hydrogen, **c** ethanol, **d** pH, and **e** acetate. *Error bar* represents the standard deviation from three independent incubation batches

Figure 1 clearly indicates that pretreatment altered the hydrolysis and acidogenesis efficiency of the straw. It is noted that the values of DOC, ethanol, acetic acid, and H_2_ of the control batch were subtracted from those of the experimental samples. The maximal DOC yields of the straw substrates were 179 ± 8, 159 ± 5, 315 ± 23, 337 ± 5, and 368 ± 14 g-C/g-DW (Note: DW means a dry weight basis), or 238 ± 11, 213 ± 7, 409 ± 29, 404 ± 6, and 411 ± 16 g-C/g-holocellulose on the basis of summing cellulose and hemicellulose, for S, DS, PS2, PS3, and NS samples, respectively. Therefore, the hydrolysis efficiency of the straw did not improve upon dilute sulfuric acid pretreatment, whereas it was increased by 76–88% on a dry weight basis with PAA pretreatment and further increased by 106% with a combination of PAA and alkali pretreatment.

However, the ultimate yields of acidogenesis metabolites were improved to a lesser extent. In fact, dilute sulfuric acid pretreatment decreased the ultimate yields of H_2_, ethanol, and acetic acid by 31.0, 0.5, and 37.4%, respectively. PAA pretreatment increased the ultimate yields of ethanol by 15.5 and 12.5% with treatment for 2 and 3 h, but decreased the yields of H_2_ by 21.8 and 29.3% and decreased those of acetic acid by 26.6 and 15.9%, respectively. Only the NS pretreatment increased the ultimate yields of H_2_ and ethanol by 16.3 and 45.5%, respectively, and decreased that of acetic acid by 9.5%. Overall, the carbon conversion yield of liquid metabolites (including ethanol and acetic acid) increased by 13.5% for NS and decreased by 21.9, 9.0, and 4.0% for DS, PS2, and PS3, respectively.

Nevertheless, the acidogenesis rates were accelerated during the first 60 days except for DS. The maximum generation rates, in the order of S, DS, PS2, PS3, and NS, were 0.041, 0.029, 0.057, 0.058, and 0.062 mmol/(g-DW h) for H_2_ (Fig. 1b); 0.50, 0.70, 0.73, 0.80, and 0.97 mg-C/(g-DW h) for ethanol (Fig. 1c); and 0.47, 0.30, 0.54, 0.55, and 0.66 mg-C/(g-DW h) for acetic acid (Fig. 1e), respectively. As a result, the pH decreased sharply for PS2, PS3, and NS (Fig. 1d). However, NS pretreatment obviously enhanced the buffer capacity.

In order to understand the yields of metabolites and the shifts in the metabolic pathway produced by substrate pretreatment, the measurements discussed in the following sections focused on the characterization of the properties of substrates after different pretreatments.

### Biochemical compositions and specific surface areas of substrates

Table 1 shows the biochemical compositions and specific surface areas of untreated and pretreated rice straw. As expected, dilute acid pretreatment removed almost all hemicellulose as well as some ash and lignin, as indicated by the increased ratio of cellulose to lignin (3.44 compared with 3.08 of S). PAA pretreatment removed almost all lignin and some hemicellulose, while increasing the pretreatment time from 2 to 3 h only removed further hemicellulose but not lignin. Alkali treatment after PAA treatment could further reduce the content of ash and hemicellulose.

**Table 1:** Biochemical components and specific surface area of rice straw

Parameter	Unit	S	DS	PS2	PS3	NS
Biochemical components						
Hemicellulose	g/g-DW	0.346 ± 0.000	0.022 ± 0.000	0.111 ± 0.005	0.071 ± 0.001	0.085 ± 0.003
Cellulose	g/g-DW	0.406 ± 0.002	0.725 ± 0.003	0.659 ± 0.005	0.762 ± 0.001	0.811 ± 0.003
Lignin	g/g-DW	0.132 ± 0.005	0.211 ± 0.000	0.031 ± 0.001	0.037 ± 0.000	0.029 ± 0.003
Ash	g/g-DW	0.067 ± 0.014	0.036 ± 0.002	0.068 ± 0.001	0.076 ± 0.000	0.015 ± 0.002
Specific surface area						
BET	m^2^/g-DW	0.887	8.021	1.899	1.911	0.793
Langmuir	m^2^/g-DW	1.409	11.995	2.844	2.875	1.224

The specific surface area determined by the Langmuir equation was about 1.5–1.6 times the value determined by the BET equation. Nevertheless, both methods show the same trend, namely that the specific surface area increased eightfold after dilute acid pretreatment, doubled after PAA pretreatment, and was reduced by 10% by NS pretreatment.

Specific surface area is usually regarded as being positively correlated with the accessibility to holocellulose [3]. Unfortunately, the present study demonstrated the reverse phenomenon, i.e., the substrate after NS pretreatment possessed less surface area but had the highest valorization efficiency. In comparison, although dilute acid pretreatment improved the surface area greatly, it did not enhance the subsequent fermentation. Although hemicellulose and lignin are the two components that are regarded as limiting the accessibility to cellulose [3], the reduction in the hemicellulose limitation after dilute acid treatment did not improve hydrolysis or acidogenesis, whereas the reduction in the lignin limitation after PAA treatment did improve hydrolysis. However, the enhancement of acidogenesis contributed more to cellulose modification in the NS treatment.

### Micro-morphological structure of straws before and after fermentation

**Fig. 2:**
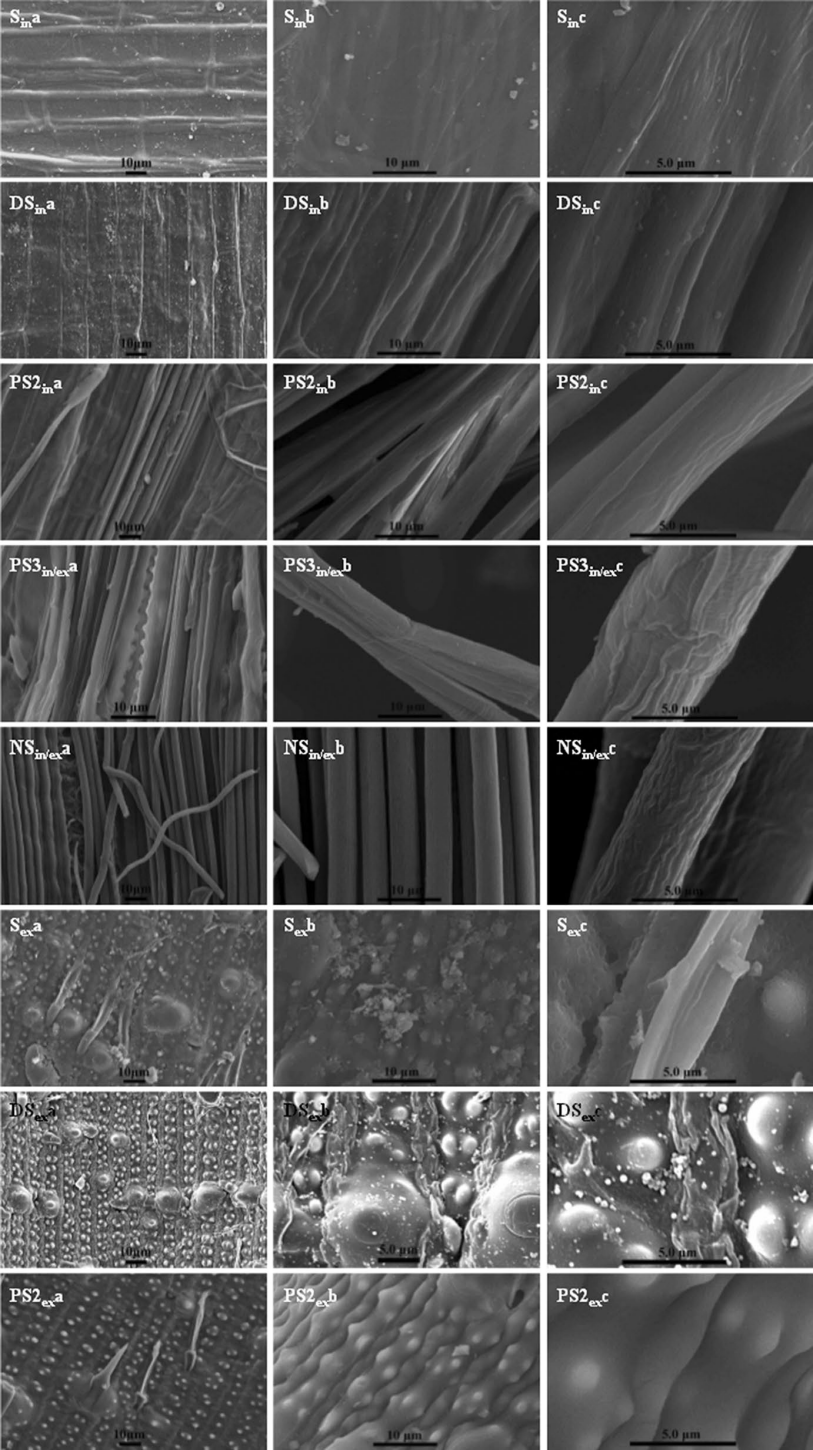
SEM images of the interior and exterior surfaces of straw before fermentation. *In* interior; *Ex* exterior; *In*/*Ex* too thin to be distinguished from the interior or exterior surface

The interior and exterior surfaces of straws were observed by SEM before fermentation (Fig. 2). As noted, for PS3 and NS, the straw pieces were too thin to allow distinction between the interior and exterior surfaces. As expected, in the order of S, DS, PS2, PS3, and NS, the epidermis was increasingly exfoliated. In the case of the DS interior surface, vascular bundles were exposed to some extent but were still connected by cuticle and parenchyma, and covered with some lignin droplets. Amounts of vascular bundles were clearly observed for PS-treated straws, but longer treatment times led to increased roughness of the bundles. For NS, the fiber bundles with diameters of 3–5 µm were completely liberated, and some were fractured, and more cellulose fibers with a diameter of 0.2–0.5 µm appeared on the bundles, leading to increased roughness of the bundles. As to the exterior surface, DS pretreatment led to the exfoliation of the epidermis and then the appearance of nodules and a mastoid structure; furthermore, lignin droplets were regenerated during dilute acid pretreatment [28]. In contrast, the exterior surface of PS2 straw had more vascular bundles exposed, the nodules and mastoid structure were partially dissolved, and the lignin droplets were not observed.

**Fig. 3:**
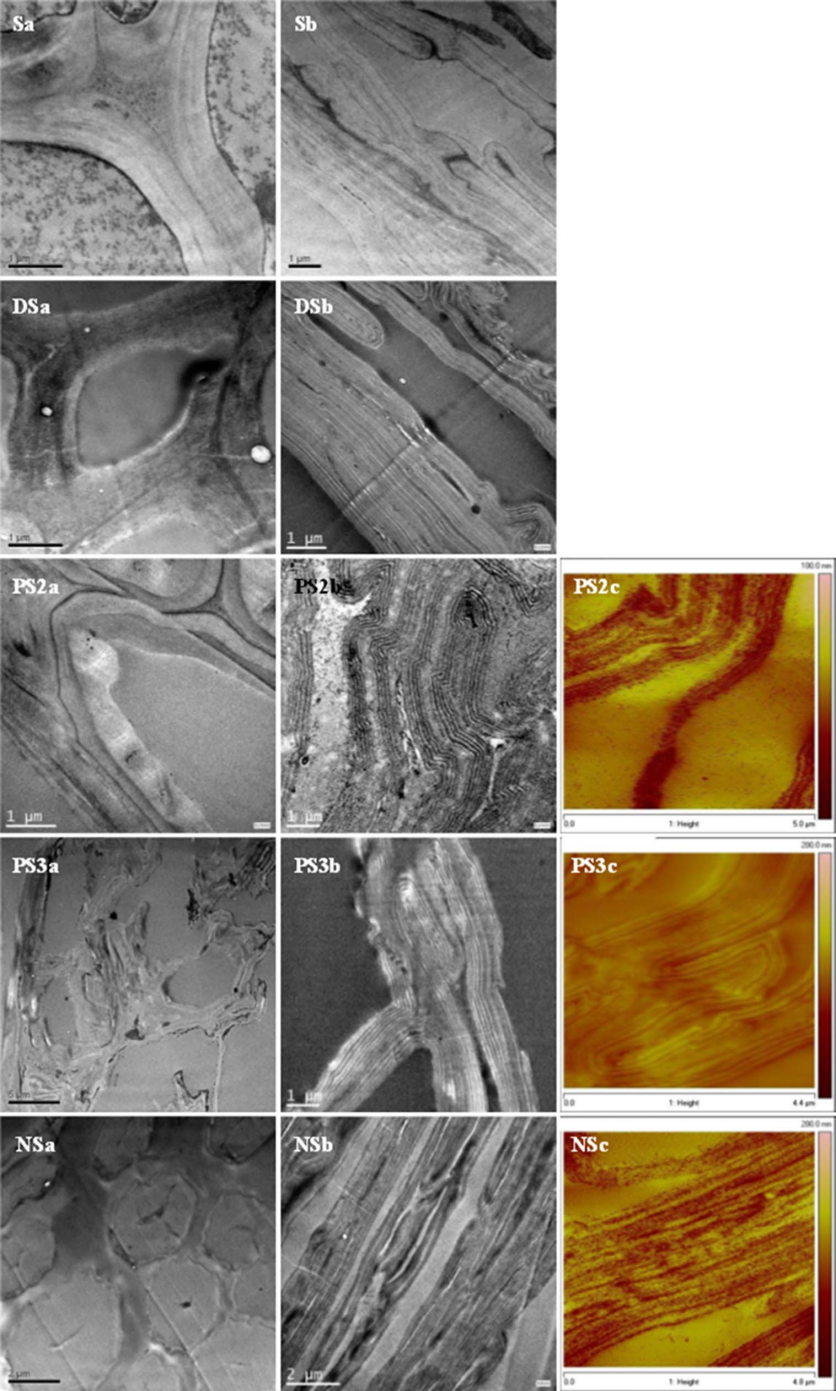
TEM and AFM images of straw before fermentation. PS2c, PS3c, NSc: AFM images

Slices of straws were observed by TEM and AFM (Fig. 3). The cell wall of the untreated straw was composed of sheets of parenchyma cells and bundle sheath cells that have a compact structure. Dilute acid pretreatment resulted in the appearance of pores, looseness of the walls, and obvious shrinking of the secondary cell walls. PAA pretreatment resulted in the removal of substances in intercellular layers and lumens, and the fracture of bundle sheath shells. Further NS pretreatment resulted in the severe shrinking of bundle sheath cells and complete destruction of the original structure, i.e., the structural substances were repartitioned. Meanwhile, the roughness estimated from AFM images suggested that NS pretreatment led to the greatest roughness, i.e., the maximum, average, and root mean square values were 175, 15.2, and 18.6 nm, while those of the PS2 sample were 93.9, 8.13, and 10.6 nm, and those of the PS3 sample were 71.1, 5.95, and 7.56 nm, respectively.

**Fig. 4:**
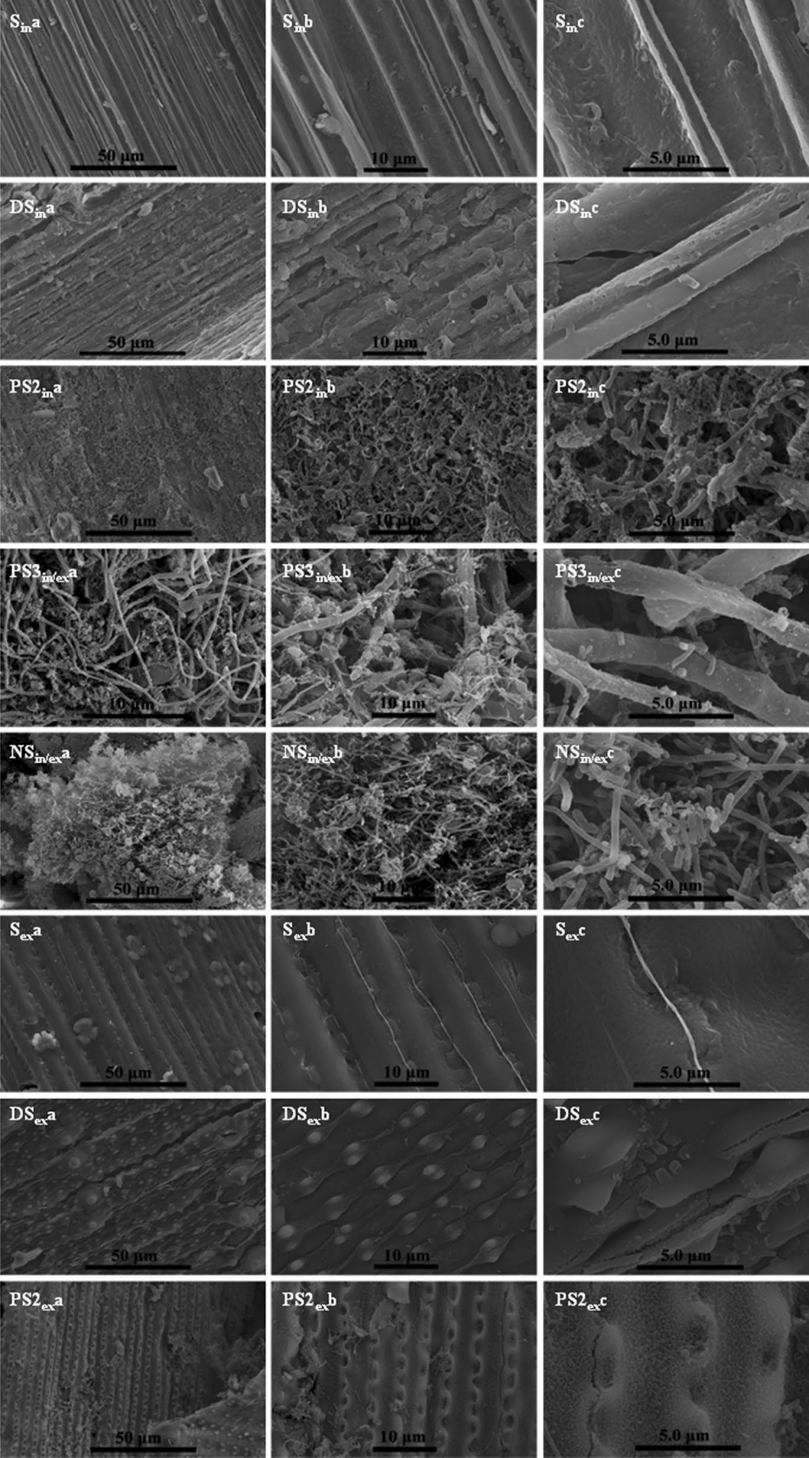
SEM images of the interior and exterior surfaces of straw after fermentation. *In* interior; *Ex* exterior; *In*/*Ex* too thin to be distinguished from the interior or exterior surface

The corresponding SEM images of straw samples after fermentation are shown in Fig. 4. Bacterial degradation damaged the epidermis of S straw, revealing abundant bundles and a porous structure. For DS, the bundles and pores were recovered or refilled by fragments. The bare bundles of PS2 straw were greatly destroyed and covered by *C. thermocellum* cells. In the case of PS3, the bundles were dispersed with the diameters being reduced to 0.5–1.5 µm. For the NS sample, no cellulose fibers could be observed in the residue, which was spongy and full of *C. thermocellum* cells. Although the exterior surfaces of the straw were highly recalcitrant to bacterial attack, they showed similar exposure of bundles and some fracturing of nodules and mastoid structure after fermentation. Therefore, *C. thermocellum* utilized the straw in a processive way from outside gradually to inside.

### Functional groups on the lignocellulosic surface

**Fig. 5:**
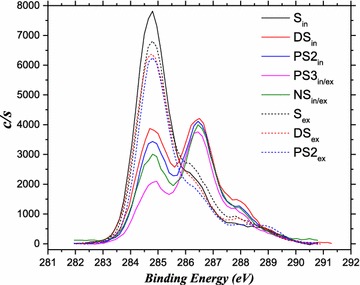
XPS C1s spectrum of straws. *In* interior; *Ex* exterior; *In*/*Ex* too thin to be distinguished from the interior or exterior surface

The carbon in cellulose is bound to O by C–O or C=O bonds, while C–C bonds mainly appear in hemicellulose, lignin, and other contaminants. Therefore, the relative proportion of C–C bonds in lignocellulose can represent the level of cellulose contamination by lignin or hemicellulose [29, 30]. The C1s zone of the XPS spectrum in the range of 282–291 eV binding energy displayed four peaks corresponding to the bonds of C–(C,H) (284.8 ± 0.1 eV), C–O (286.3 eV), C=O(O–C–O) (288.2 ± 0.2 eV), and COOH (289.0 eV) (Fig. 5). To avoid peak overlap and to identify the individual contributions of each bond, the peaks were fitted using XPS PEAK 4.1 software. The results of spectrum fitting are shown in Table 2. There are minor differences in the values for exterior surfaces among S, DS, and PS2. In contrast, the distribution of bonds across the interior surfaces was greatly altered by pretreatments. Compared to S, the proportion of C–(C,H) bonds was reduced by 41.3, 46.8, 57.6, and 47.0%, respectively, in DS, PS2, PS3, and NS. C–O bond proportions were increased by 120–175% and those of C=O(O–C–O) bonds were increased by 145–183%. Although the proportion of COOH bonds was reduced by 7–55%, their original proportion in S was low at 4.09%. Therefore, all four pretreatments effectively removed the constraints of hemicellulose or lignin on cellulose accessibility. Compared with PAA alone, the subsequent alkali pretreatment had a negligible effect on hemicellulose and lignin.

**Table 2:** Functional groups of the lignocellulosic surface

Samples	Proportion of bonds (%)				O/C atomic ratios		Ratio of C–O to [C=O(O–C–O) + COOH]
	C–(C,H)	C–O	C=O(O–C–O)	COOH			
Interior							
S_in_	71.42	19.05	5.43	4.09	0.63		2.00
DS_in_	41.93	41.83	14.00	2.24	1.25		2.58
PS2_in_	37.99	44.92	13.30	3.79	1.34		2.63
PS3_in_	30.25	52.39	13.70	3.67	1.49		3.02
NS_in_	37.84	44.95	15.38	1.83	1.27		2.61
Exterior							
S_ex_	67.04	21.98	8.03	2.95	0.86		2.00
DS_ex_	69.32	19.31	7.10	4.28	0.82		1.70
PS2_ex_	67.40	21.64	5.67	5.29	0.67		1.97

Higher values of the atomic ratio of O/C or the ratio of C–O bonds to [C=O(O–C–O) + COOH] bonds can indicate higher contents of cellulose. The values of both indicators were slightly reduced for the exterior surfaces and significantly increased for the interior surfaces by pretreatments. Noticeably, the values for NS were lower than those for PS2 and PS3, although the biochemical components suggested higher cellulose content (Table 1). This implies that the increased cellulose content could mainly be attributed to decreased ash.

### Modification of cellulose biomolecules by pretreatments

**Fig. 6:**
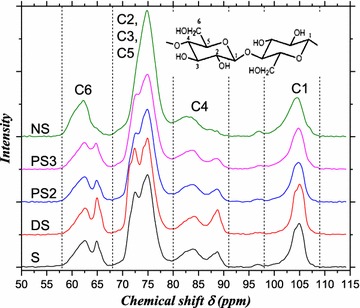
CP/MAS ^13^C NMR spectrum of straws

Figure 6 shows the CP/MAS ^13^C NMR spectra of straw fibers. The positions of carbon atoms in a glucose unit of cellulose are marked as C1–C6 (Fig. 6). The chemical shifts, *δ*, may be assigned as follows: C1 (98–109 ppm), C4 (80–91 ppm), C2/C3/C5 (68–80 ppm), and C6 (58–68 ppm). For C6, *δ* in the 64.5–66.0 ppm region originates from type I cellulose. For C1, *δ* in the 106–107 ppm region originates from type II cellulose. To avoid peak overlaps and to identify the characteristic peaks, spectrum fitting was applied using the software MestReC-V 4.9.9.9 for the spectral regions of C1 and C6. The fitting results are listed in Table 3. Since *δ* in the 80–86 ppm region originates from amorphous cellulose and *δ* in the 86–91 ppm region originates from crystalline cellulose, the crystallinity index CrI is defined as the ratio of the peak area in the 86–91 ppm region to that in the 80–91 ppm region. The CrI values are also listed in Table 3.

**Table 3:** Proportion of different crystalline cellulose and the CrI of cellulose

Crystalline types	Chemical shift (ppm)	Proportion (%)				
		S	DS	PS2	PS3	NS
Cellulose I (C6)	64.5–66.0	28.1	36.9	27.4	32.0	7.91
Cellulose II (C1)	106.0–107.0	ND^a^	ND^a^	ND^a^	ND^a^	11.8
Cellulose *I*_*α*_ (C1)	104.9 (0.1)	62.5	59.6	43.7	35.1	8.90
Cellulose *I*_*β*_ (C1)	105.9, 103.8	15.8	26.8	32.2	51.9	49.5
Less-ordered (C1)	103.2, 102.6 (0.2) 21.7	21.7	13.6	24.1	13.0	27.8
Crystallinity index CrI		35.4	45.8	32.8	29.6	16.8

^a^ *ND* Not detected

The most significant changes appeared for NS pretreatment, where the proportion of type I cellulose decreased from 28.1% in S to 7.91% in NS, while the undetected type II cellulose in S increased to 11.8% in NS. Furthermore, the ratio of *I*_*β*_–*I*_*α*_ increased upon pretreatment, i.e., to 0.25, 0.45, 0.74, 1.48, and 5.56, respectively, for S, DS, PS2, PS3, and NS. Thus, NS pretreatment modified type I cellulose to type II [16] and other crystalline or amorphous celluloses. As cellulose II has more amorphous regions than cellulose I [31], it is understandable that CrI of NS was reduced by 53%. Comparatively, DS increased the proportion of type I cellulose by 31% and CrI by 29%. PAA pretreatment increased CrI by only 7–16%.

The degree of polymerization of the cellulose was assessed by the indicators of weight-average DP_w_ and weight-average DP_n_. As listed in Table 4, DS decreased DP_w_, DP_n_, and polydispersity the most. PAA pretreatment for 2 h decreased the values of the above three parameters, while longer operation resulted in a gradual increase in the values. NS conversely increased DP_n_ and the polydispersity, implying that NS depolymerized cellulose and then polymerized it again.

**Table 4:** Polymerization degree of cellulose

Polymerization degree	S	DS	PS2	PS3	NS
DP_w_	190	58	152	153	168
DP_n_	553	115	375	440	776
Polydispersity	2.91	1.99	2.47	2.87	4.61

### Biodegradability of substrates and residues

**Fig. 7:**
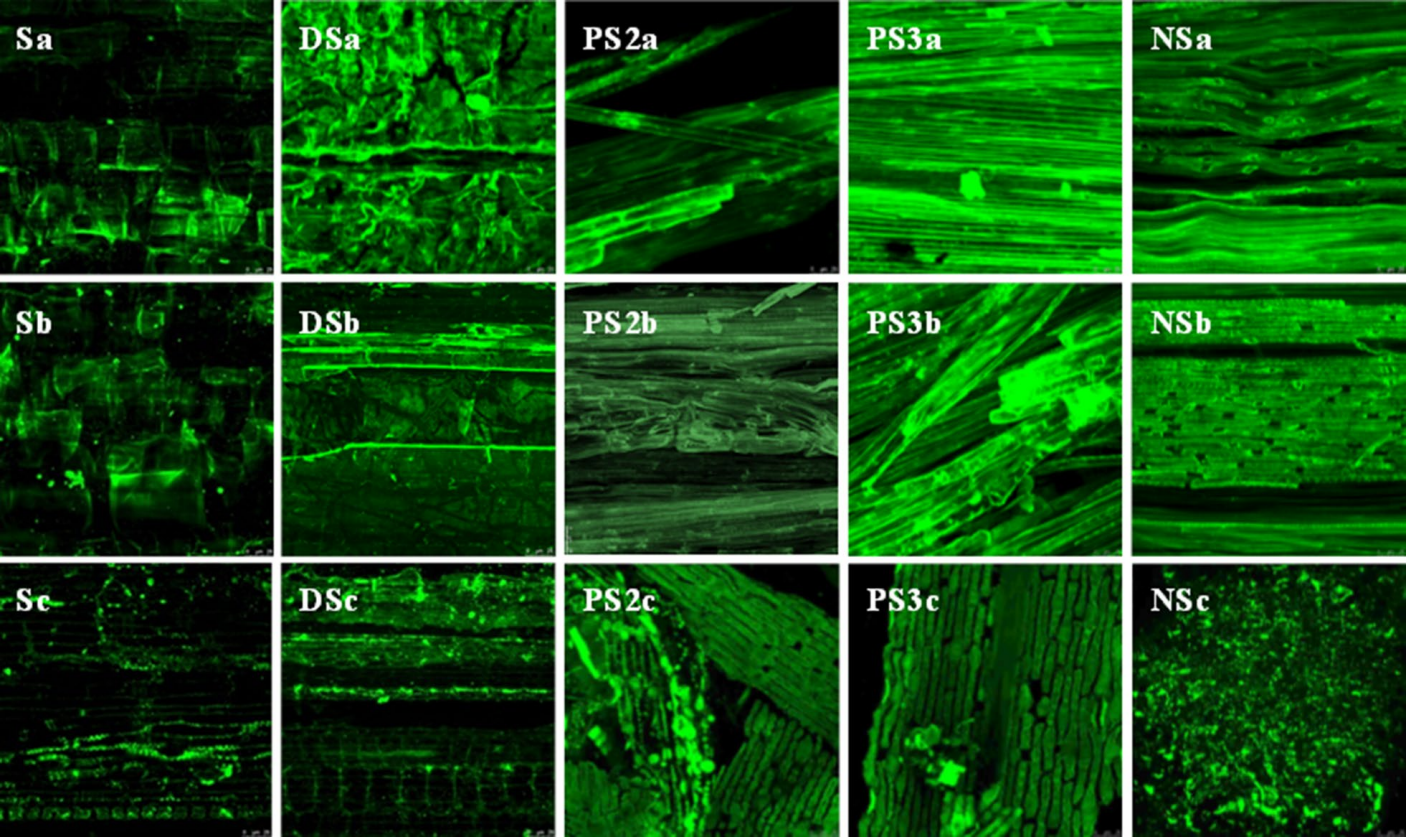
TGC labeling on lignocellulosic surfaces: **a** straw before fermentation, without BSA blocking; **b** straw before fermentation, with BSA blocking; and **c** straw after fermentation, with BSA blocking

Since TGC labeling utilizes the binding of fluorescent cellulose-binding module 3 (CBM3) protein to lignocellulosic material, the green fluorescence intensity implies the proportion of the surface accessible to CBM3. The increased fluorescence intensities of the pretreated substrates in Fig. 7b indicate that more accessible surfaces were exposed by the pretreatments, and especially by PS3 and NS. The difference between Fig. 7a without BSA blocking and Fig. 7b with BSA blocking indicates the contribution from non-productive binding to lignin. Therefore, if estimated according to the mean fluorescence intensity, 38% of the CBM3-accessible surface was non-productive owing to the sediment of lignin droplets. Comparatively, the fluorescence differences between Fig. 7a and Fig. 7b for PS2, PS3, and NS were not evident, suggesting that the contribution of lignin could be ignored in these pretreated substrates. Meanwhile, the adhesive mass among vascular bundles in DS disappeared after PAA pretreatment. As a result, the images revealed that the closely packed vascular bundles of PS3- and NS-pretreated substrates were all covered by green proteins, and fractures in the bundles were obvious for NS.

The remaining residues after straw fermentation were still fluorescent to different extents (Fig. 7c with BSA blocking). S and DS residues displayed lower fluorescent intensities than the substrates but still retained a similar morphology. PS residues showed green fractured bundles that should theoretically be accessible to cellulase. As for NS, only some debris remained.

## Discussion

### Influence of pretreatment on hydrolysis

The average carbon contents of cellulose and rice straw are about 41.8% DW [32] and 48.7% DW [33], while the maximal DOC yields reached 368 ± 14 g-C/g-DW for NS (Fig. 1). Considering that 21–27% of the cellobiose will be used for biosynthesis to give biomass and extracellular enzymes [34], the hydrolysis efficiency of NS-pretreated straw was thus close to 100%. Compared with PS3-pretreated straw, whose lignin and hemicellulose contents (Table 1) and accessibility (Fig. 7) were roughly equivalent to those of NS, a 10% increment in DOC indicated that the hydrolysis efficiency for NS could be attributed to the modification of cellulose from type I to type II and from *I*_*α*_ to *I*_*β*_ (Table 3), its lower CrI (Table 3) and smaller diameter and fracture of its vascular bundles (Figs. 2, 3), and the reduction in the amount of ash (Table 1). Since cellulose II has more accessible crystal surfaces [31] and amorphous regions [31], lower CrI [18] and smaller nanowhiskers [35] than cellulose I, the digestibility evaluated by cellulase [11, 16–18] is higher for type II than type I cellulose. Meanwhile, the reduction in silica components (as suggested by the reduced ash content for NS) can improve enzymatic hydrolysis [36]. Furthermore, alkaline pretreatment can modify lignin by cleaving β-*O*-4 linkages, resulting in the formation of new phenolic hydroxyl groups, which leads to a substantial decrease in the molecular mass of the residual lignin and a more hydrophilic character [37]. Alkaline pretreatment can also remove the acetyl groups of hemicellulose [38]. As noted, the hydrolysis benefit from the additional alkaline treatment was not significant at the beginning because the DOC generation rates were nearly equal for PS2, PS3, and NS.

Delignification by PAA seemed to be the most effective means of enhancing the hydrolysis efficiency (Fig. 1; Table 1), where the maximal DOC yields of PS2 and PS3 were 76 and 88%, respectively, higher than that of S. In comparison, although dilute sulfuric acid removed almost all of the hemicellulose (Table 1), expanded the specific surface area eightfold (Table 1), and depolymerized the cellulose to a quarter degree (Table 4), DS pretreatment led to the formation of sediments of lignin droplets (Figs. 2, 3), increased the proportion of type I cellulose, and increased the cellulose CrI (Table 3). As a result, the productive CBM3 accessibility (Fig. 3) and bacterial hydrolysis efficiency (Fig. 1) were not improved by DS pretreatment.

### Properties of cellulose affect the metabolic pathway and efficiency

With regard to lignocellulosic feedstock, their theoretical ethanol yields are usually calculated from the hemicellulose and cellulose contents [39]. It is understandable that the fermentation efficiency of a 5-carbon hemicellulose is different from that of a 6-carbon cellulose [40, 41]. *C. thermocellum* expressed different cellulosomal patterns [42] and global gene patterns [43, 44] when grown on either cellulose or cellobiose, corresponding to the slightly varied ratios of ethanol to acetate products when grown on cellulose, cellobiose, or cellodextrin with different degrees of polymerization [45]. The ethanol potential of lignocellulosic biomass after pretreatment varies and is difficult to predict owing to interactions between several factors [3]. However, to the best of our knowledge, no previous report has definitively pointed out that the crystalline form of cellulose can alter the fermentation pathway. Again, taking NS- and PS3-pretreated straws for comparison, the ultimate ethanol, H_2_, and acetate yields of NS were, respectively, 29, 65, and 8% higher than that of PS3 (Fig. 1), which could not be attributed to the 10% increment in hydrolysis efficiency. Obviously, NS dominated by cellulose II and *I*_*β*_ was more favorable for H_2_ and ethanol production than PS3 dominated by cellulose *I* and *I*_*α*_. Therefore, the crystalline form altered the susceptibility of cellulose to bio-oxidation. In contrast, Carlsson et al. [46] observed that *I*_*α*_- and *I*_*β*_-dominated cellulose did not differ in susceptibility to oxidation by 2,2,6,6-tetramethylpiperidine-1-oxyl. Hence, cellulose transformation should be another important driving force for enhancing the production rates and yields of H_2_ and methanol.

### Pretreatment led to an increase in low-value by-products or pollutants

Notably, at the end of 900 h of fermentation, a portion of the hydrolysates remained unused by *C. thermocellum*, indicated by the fact that the carbon contents of ethanol and acetic acid could only cover 82% of DOC for S, 68% for DS, and 43–45% for PS2, PS3, and NS. (Note: other acids were detected in small amounts; data not shown). Lynd et al. [47] noticed that for many studies the carbon balance was not close and indeed was <70% when accounting for commonly measured fermentation products of *C. thermocellum* incubation. The authors further determined that 11.1% of the unbalanced carbon in the liquids originated from malate, pyruvate, uracil, soluble glucans, and extracellular free amino acids for a 60-h *C. thermocellum* incubation [47]. Carbon flux distribution for cellulose fermentation by *C. cellulolyticum* [48, 49] and the metabolome of *C. thermocellum* [50] supported the generation of extracellular proteins/polysaccharides/pyruvate and free amino acids in large quantities.

Substrate availability could affect the gene expression of *C. thermocellum* and might therefore regulate the metabolism, because the composition of cellulosome and other hydrolytic enzymes in *C. thermocellum* has been found to be dependent on carbon sources (dilute acid-pretreated switchgrass, cellobiose, amorphous cellulose, crystalline cellulose, and combinations of crystalline cellulose with pectin and/or xylan [51]; crystalline cellulose and non-cellulosic substrates, low substrate availability [52]). It has been reported that the cellulosomal genes are regulated by the presence of extracellular polysaccharides via an external carbohydrate-sensing mechanism [53]. As a result, a series of metabolic by-products, which are not usually associated with *C. thermocellum* fermentation, including malate, pyruvate, uracil, soluble glucans, and extracellular free amino acids [47], could be detected at different extents. Furthermore, exposure to ethanol could further change the transcriptomics, proteomics, and metabolomic profiles of *C. thermocellum* [54].

That is to say, although pretreatment may improve the hydrolysis efficiency to different extents, the hydrolysates produced in increased amounts are not converted to the targeted ethanol or acetate, but to unfavorable substrates for the selected consortium or to low-value by-products, which are difficult resources to value, and finally have to be regarded as wastewater. As a result, the benefits afforded by the improved digestibility following pretreatment are severely reduced by the added requirement for wastewater treatment. Even acetate is difficult to separate and use, owing to its high hydrophilicity, which leads to its limited applications as feedstock for methanization, as a carbon source for biological nutrient removal from wastewater [55], or as a participant in chain elongation to medium-chain carbon products [56].

Compared with S, ethanol production was increased by 15.5, 12.5, and 45.5%, respectively, through PS2, PS3, and NS pretreatment. However, the aqueous non-ethanol DOC production was increased by 104, 122, and 136%, resulting in a huge burden for wastewater treatment.

Additional file 1 shows a sketch diagram of the effect of precise pretreatment on substrate characteristics, hydrolysis, fermentation, and trade-off between energy benefits and pollution.

## Conclusion

The transformation of cellulose crystalline forms from I to II and from *I*_*α*_ to *I*_*β*_ is essential to improving the production of H_2_ and ethanol. Meanwhile, pretreatment not only increased the yield of the targeted products, but also expanded the yield of unwanted by-products to a greater extent. Therefore, the effectiveness of pretreatment should not only be determined according to energy auditing and effects of the accompanying inhibitors, but should also be assessed in terms of the environmental benefits of the whole integrated system.

## Additional file

**Additional file 1.** Graphic Abstract. It contains a sketch diagram about the effect of pretreatment on hydrolysis, fermentation and potential pollution.

## Abbreviations

BSA: bovine serum albumin
CBM3: cellulose-binding module 3
DOC: dissolved organic carbon
DS: dewaxed rice straw pretreated with dilute sulfuric acid
DW: dry weight
NS: dewaxed rice straw pretreated with peracetic acid and then NaOH
PAA: peracetic acid
PS: dewaxed rice straw pretreated with peracetic acid, PS2 for 2 h, and PS3 for 3 h
S: dewaxed rice straw
TGC: Trx-green fluorescent protein-CtCBM3
VS: volatile solid
wt: weight
