# Supplementary material for: Precise pretreatment of lignocellulose: relating substrate modification with subsequent hydrolysis and fermentation to products and by-products
Source: Biotechnol Biofuels. 2017 Apr 11;10:88. doi: 10.1186/s13068-017-0775-3 (PMC5387280; doi:10.1186/s13068-017-0775-3)
Supplement: Supplementary file 1 — Additional file 1. Graphic Abstract. It contains a sketch diagram about the effect of pretreatment on hydrolysis, fermentation and potential pollution. [file 13068_2017_775_MOESM1_ESM.docx]

**Additional file 1:**

**Graphic Abstract**

More effective the pretreatment was for ethanol production, more non-ethanolic low-value by-products were produced, namely, intensifying environmental burden.
